# Left ventricular decompression on Veno-arterial extracorporeal membrane oxygenation with intra-aortic balloon Counterpulsation

**DOI:** 10.1186/s13019-019-0970-3

**Published:** 2019-08-22

**Authors:** Jan M. Griffin, Susan Restaino, Koji Takeda, Arthur R. Garan

**Affiliations:** Columbia University Irving Medical Center, 173 Fort Washington Avenue, Room 4617, 630 West 168th St, New York, NY 10032 USA

**Keywords:** Acute heart failure, Extracorporeal circulation, Intra-aortic balloon pump, Rejection, Transplant

## Abstract

**Background:**

Veno-arterial extracorporeal membrane oxygenation (VA-ECMO) has been used increasingly to support patients with cardiogenic shock (CS). There has been growing recognition of the favorable and unfavorable hemodynamic effects of this therapy and recent interest in the use of other percutaneous circulatory support devices to offset some of the potentially harmful hemodynamic effects. Herein, we provide visual evidence of the effects of intra-aortic balloon pump (IABP) counterpulsation for a patient with peripheral VA-ECMO cannulation.

**Case presentation:**

A 68 year old man who had undergone orthotopic heart transplantation presented with 2 days of fatigue, orthopnea, and paroxysmal nocturnal dyspnea. On examination, he was tachycardic, hypotensive and hypoxic with cool extremities, consistent with CS. Transthoracic echocardiogram (TTE) showed new severe biventricular dysfunction with a left ventricular ejection fraction of 15%, right heart catheterization demonstrated elevated filling pressures and low output. An IABP was inserted via the left femoral artery with minimal improvement in hemodynamics. He was escalated to VA-ECMO. Repeat TTE demonstrated aortic valve (AV) opening with each cardiac cycle and mild MR. With placement of the IABP on standby Additional file 1: Video 1 (video 0:03), the AV no longer opened. Re-initiation of balloon counterpulsation resulted in resumed AV opening with each beat Additional file 1: Video 1 (video 0:17). He was treated for presumed acute allograft rejection with methylprednisolone, thymoglobulin, intravenous immunoglobulin and plasmapheresis with improvement in allograft function. However, he developed an *Enterobacter aerogenes* pneumonia and rapidly fatal septic shock.

**Conclusions:**

This case visually demonstrates effective LV decompression by IABP counterpulsation in VA-ECMO support. While the overall effects of LV decompression in patients on VA-ECMO with IABP are still unclear, this report demonstrates one potential mechanism of benefit in the prevention of stagnation of blood flow that may lead to intra-cardiac or aortic root thrombus formation.

**Electronic supplementary material:**

The online version of this article (10.1186/s13019-019-0970-3) contains supplementary material, which is available to authorized users.

## Background

An increasingly well-recognized effect of peripherally inserted veno-arterial extracorporeal membrane oxygenation (VA-ECMO) in patients with cardiogenic shock (CS) is that of retrograde aortic flow and the resultant additional afterload on the already failing left ventricle (LV) [1]. This can cause increased LV end-diastolic pressure (LVEDP) and thus LV distension (LVD) which may be associated with impaired myocardial recovery or other signs of LV pressure overload such as ventricular arrhythmia, pulmonary edema or intracardiac thrombus formation [2, 3]. In such cases, unloading of the LV may prevent these complications or even facilitate ventricular recovery. Herein, we provide visual evidence of the effects of intra-aortic balloon pump (IABP) counterpulsation for a patient with peripheral VA-ECMO cannulation.

## Case presentation

A 68 year-old man with a history of hypertension, diabetes mellitus and heart transplantation due to ischemic cardiomyopathy presented with 2 days of fatigue, orthopnea, and paroxysmal nocturnal dyspnea. On examination, he weighed 78 kg (body surface area 1.9m^2^), he was tachycardic, hypotensive (mean arterial pressure [MAP] 57 mmHg) and hypoxic with cool extremities, consistent with CS. Dopamine was initiated at 10mcg/kg/min. Transthoracic echocardiogram (TTE) showed new severe biventricular dysfunction with LV ejection fraction of 15%. He was brought to the cardiac catheterization lab for endomyocardial biopsy and right heart catheterization (RHC) which demonstrated elevated filling pressures (right atrial pressure (RAP) 22 mmHg, mean pulmonary artery pressure (mPAP) 33 mmHg, pulmonary capillary wedge pressure (PCWP) 36 mmHg, pulmonary artery saturation (PA sat) 43%, and Fick cardiac index 1.5 L/min/m^2^). An IABP was inserted via the left femoral artery. Balloon counterpulsation provided minimal improvement in his hemodynamic profile (PA sat 38%). As a result, VA-ECMO was initiated via the right femoral artery and vein with flow of 3.0 L/min (1.6 L/min/m^2^). Repeat TTE demonstrated aortic valve (AV) opening with each cardiac cycle. With placement of the IABP on standby Additional file 1: Video 1 (video clip 0:03), the AV no longer opened. Reinitiation of balloon counterpulsation resulted in resumed AV opening with each beat Additional file 1: Video 1 (video clip 0:17). Repeat hemodynamics showed MAP 65 mmHg, RAP 10 mmHg, mPAP 27 mmHg, PCWP 22 mmHg and PA sat 56%, dopamine was discontinued. He was treated for presumed acute allograft rejection with methylprednisolone, thymoglobulin, intravenous immunoglobulin and plasmapheresis. With this his hemodynamics improved; VA-ECMO was removed after 11 days and IABP was removed 3 days later (hemodynamics: RAP 13 mmHg, mPAP 32 mmHg, PA sat 53%). Repeat TTE showed improvement in allograft function. However, he developed an *Enterobacter aerogenes* pneumonia and rapidly fatal septic shock.

## Discussion and conclusions

This case visually demonstrates effective LV decompression by IABP counterpulsation in VA-ECMO support. Due to severe allograft failure, the AV remained closed during hemodynamic support with VA-ECMO alone as a result of pressure overload created by the retrograde aortic flow. In contrast, as an adjunctive therapy, the IABP facilitated opening of the AV likely by reducing afterload and diminishing the pressure differential across the valve. The intra-cardiac filling pressures during concomitant VA-ECMO and IABP support were reduced and overall cardiac output augmented significantly. In the absence of IABP counterpulsation the patient would have likely developed pulmonary edema and either intra-cardiac or aortic root thrombus.

VA-ECMO has been utilized with increasing frequency for patients suffering from CS [4]. However, when peripherally cannulated, it can impose additional workload on the already failing LV due to retrograde aortic flow, increasing ventricular afterload and subsequently elevating LVEDP [1]. This LV pressure overload may manifest as pulmonary edema or ventricular arrhythmia. In extreme cases such as this one, the AV may remain closed resulting in stagnation of blood. Between 10 to 68% of adult and pediatric patients develop signs of LV pressure overload or LVD on VA-ECMO [5, 6]. However, there are no guidelines to assist with predicting who will develop LVD post VA-ECMO cannulation. Currently, whether to proceed with LV decompression is at the discretion of the treating team and based on several factors including clinical, hemodynamic, echocardiographic and radiographic parameters. In addition, there is no clearly superior means of LV decompression. Options include IABP, percutaneous LV assist device (such as Impella), atrial septostomy, and direct LV cannulation. Retrospective studies have demonstrated some benefits to use of these strategies to either prevent overt signs of pressure overload or facilitate ventricular recovery [3, 7].

Several small studies have investigated the combination of IABP with VA-ECMO with some demonstrating a clinical benefit. Aso et al. demonstrated a lower all-cause 28-day mortality and improved chance of wean from mechanical support for patients supported by VA-ECMO and IABP as opposed to VA-ECMO alone [7]. In addition, Bréchot et al. demonstrated a lower risk of overt pressure overload (assessed by the development of pulmonary edema) with concomitant IABP use. The case video presented here demonstrates one additional mechanism of benefit to IABP use with VA-ECMO, namely the prevention of stagnation of blood flow that may result in intra-cardiac or aortic root thrombus formation and the devastating effects of this complication. While the overall effects of LV decompression in patients on VA-ECMO with IABP are still unclear, this report demonstrates one potential mechanism of benefit in the prevention of stagnation of blood flow that may lead to intra-cardiac or aortic root thrombus formation.

## Additional file

Additional file 1: Echo image. (MOV 1920 kb)

## Data Availability

Data sharing is not applicable to this article as no datasets were generated or analyzed during the current study.

## Abbreviations

AV: aortic valve
CS: cardiogenic shock
IABP: intra-aortic balloon pump
LV: left ventricle
LVD: LV distension
LVEDP: LV end-diastolic pressure
mPAP: mean pulmonary artery pressure
PA sat: pulmonary artery saturation
PCWP: pulmonary capillary wedge pressure
RAP: right atrial pressure
RHC: right heart catheterization
TTE: Transthoracic echocardiogram
VA-ECMO: veno-arterial extracorporeal membrane oxygenation
